# Locally Advanced Pancreatic Cancer: Work-Up, Staging, and Local Intervention Strategies

**DOI:** 10.3390/cancers11070976

**Published:** 2019-07-12

**Authors:** Eran van Veldhuisen, Claudia van den Oord, Lilly J. Brada, Marieke S. Walma, Jantien A. Vogel, Johanna W. Wilmink, Marco del Chiaro, Krijn P. van Lienden, Martijn R. Meijerink, Geertjan van Tienhoven, Thilo Hackert, Christopher L. Wolfgang, Hjalmar van Santvoort, Bas Groot Koerkamp, Olivier R. Busch, I. Quintus Molenaar, Casper H. van Eijck, Marc G. Besselink, the Dutch Pancreatic Cancer Group and International Collaborative Group on Locally Advanced Pancreatic Cancer

**Affiliations:** 1 Department of Surgery, Cancer Center Amsterdam, Amsterdam UMC, University of Amsterdam, 1105 AZ Amsterdam, The Netherlands; 2 Department of Surgery, Regional Academic Cancer Center Utrecht, University of Utrecht, 3584 CX Utrecht, The Netherlands; 3 Department of Medical Oncology, Cancer Center Amsterdam, Amsterdam UMC, University of Amsterdam, 1105 AZ Amsterdam, The Netherlands; 4 Department of Surgery, University of Colorado, Denver, CO 80045, USA; 5 Department of Radiology, Cancer Center Amsterdam, Amsterdam UMC, University of Amsterdam, 1105 AZ Amsterdam, The Netherlands; 6 Department of Radiology and Nuclear Medicine, Cancer Center Amsterdam, Amsterdam UMC, VU University, 1081 HV Amsterdam, The Netherlands; 7 Department of Radiation Oncology, Cancer Center Amsterdam, Amsterdam UMC, University of Amsterdam, 1105 AZ Amsterdam, The Netherlands; 8 Department of Surgery, Universitätsklinikum Heidelberg, 69120 Heidelberg, Germany; 9 Department of Surgery, John’s Hopkins Hospital, Baltimore, MD 21287, USA; 10 Departments of Surgery, Regional Academic Cancer Center Utrecht, St Antonius Hospital Nieuwegein, 3435 CM Nieuwegein, The Netherlands; 11 Department of Surgery, Erasmus University Medical Center, 3015 GD Rotterdam, The Netherlands

**Keywords:** locally advanced pancreatic cancer, FOLFIRINOX, explorative laparotomy, ablation, resection

## Abstract

Locally advanced pancreatic cancer (LAPC) has several definitions but essentially is a nonmetastasized pancreatic cancer, in which upfront resection is considered not beneficial due to extensive vascular involvement and consequent high chance of a nonradical resection. The introduction of FOLFIRINOX chemotherapy and gemcitabine-nab-paclitaxel (gem-nab) has had major implications for the management and outcome of patients with LAPC. After 4–6 months induction chemotherapy, the majority of patients have stable disease or even tumor-regression. Of these, 12 to 35% are successfully downstaged to resectable disease. Several studies have reported a 30–35 months overall survival after resection; although it currently remains unclear if this is a result of the resection or the good response to chemotherapy. Following chemotherapy, selection of patients for resection is difficult, as contrast-enhanced computed-tomography (CT) scan is unreliable in differentiating between viable tumor and fibrosis. In case a resection is not considered possible but stable disease is observed, local ablative techniques are being studied, such as irreversible electroporation, radiofrequency ablation, and stereotactic body radiation therapy. Pragmatic, multicenter, randomized studies will ultimately have to confirm the exact role of both surgical exploration and ablation in these patients. Since evidence-based guidelines for the management of LAPC are lacking, this review proposes a standardized approach for the treatment of LAPC based on the best available evidence.

## 1. Introduction

Pancreatic cancer is diagnosed some 340,000 times per year globally [1] and carries a 5 year cumulative survival of 5–10% [2]. Only 15–20% of patients have resectable pancreatic cancer at diagnosis and are treated with a combination of surgery and (neo)adjuvant chemotherapy. The remaining patients present with either metastatic disease (40–50%) or locally advanced, nonresectable pancreatic cancer (LAPC) due to local but extensive vascular involvement (30–40%) [3,4].

In the past decade, there have been several developments in the treatment of LAPC. First, the introduction of FOLFIRINOX (a combination therapy of leucovorin, 5-fluorouracil, irinotecan, and oxaliplatin), which on itself has led to an improvement of median overall survival from 9 to 16 months [5], and also to the possibility to convert LAPC to resectable disease in 10–35% of patients [6,7,8]. Although randomized trials are needed to confirm the benefit of surgery after FOLFIRINOX, several nonrandomized cohort studies reported a survival of 30–34 months from diagnosis for patients undergoing resection after FOLFIRINOX [9].

Determining resectability with cross-sectional imaging after FOLFIRINOX chemotherapy is, however, often troublesome [10]. Typically, computed tomography (CT) imaging will not accurately differentiate the edges of viable tumor tissue after FOLFIRINOX treatment. Due to the uncertainty in determining resectability, some authors advise to perform a surgical exploration in all patients without progression, according to the response evaluation criteria in solid tumors (RECIST) after FOLFIRINOX [11]. Nevertheless, a large number of patients will still be unresectable during surgical exploration, due to either local extent or metastases, and consequently undergo futile surgery.

Stable disease or regression (i.e., partial response) after FOLFIRINOX gives an opportunity to investigate whether further improvement is possible with local treatments, with a lower risk of performing futile interventions in patients who progress rapidly or develop metastases. This has prompted interest in both surgery and local ablative techniques in LAPC, such as irreversible electroporation (IRE), radiofrequency ablation (RFA), and stereotactic body radiation therapy (SBRT). Previous studies have shown that IRE, RFA, and SBRT are feasible techniques for the treatment of LAPC, with mostly acceptable morbidity [12] and a reported median overall survival of 23 months [13]. Hence, a multimodality approach for LAPC where patients initially are treated with chemotherapy and, in case of RECIST nonprogressive disease, proceed to an explorative laparotomy with the intention to resect or perform a local ablation when a resection is not feasible could be of major interest. The intraoperative decision to perform a resection or local ablation is, however, often difficult given the extent of both viable tumor and remaining fibrosis [10]. Surgical experience, as well as a dedicated team including specialists from pathology, interventional and diagnostic radiology, oncology, and radiotherapy within a high-volume setting appears essential for the optimal treatment of LAPC [14].

This paper describes a standardized approach for the treatment of LAPC based on the current available evidence. The authors of this review represent several clinical groups, of whom all have performed studies on resection and/or local ablation after FOLFIRINOX for LAPC. Although this is a rapidly developing field with multiple studies still ongoing, we feel it is important to present our experience, as many clinicians worldwide are confronted with patients with “nonprogressive” LAPC after induction therapy. A future international evidence-based guideline on LAPC would be a valuable next step.

## 2. Materials and Methods

### 2.1. Search

A literature search was performed using the PubMed database to identify studies reporting on the management of patients with LAPC following induction chemotherapy until January 2019 (see Appendix A for the search strategy)). In addition, the reference lists of all identified papers were searched manually to identify additional relevant studies. See Appendix A for the full search strategy.

### 2.2. Eligibility Criteria

Cohort studies (i.e., prospective and retrospective) and randomized trials published over the last 10 years in the English language were eligible for inclusion if they reported on the staging, work-up, and/or surgical management of patients with LAPC following induction chemotherapy.

### 2.3. Data Extraction

Data were extracted on patient demographics, study design, work-up, chemotherapy administration, clinical staging, biliary drainage, and surgical management.

## 3. Results

### 3.1. Clinical Staging

Staging of pancreatic cancer is usually performed using high quality CT imaging according to the National Comprehensive Cancer Network (NCCN) criteria [15], but may vary depending on local standards. According to the NCCN criteria, LAPC is defined as a pancreatic adenocarcinoma without overt distant metastases, with >180° involvement of the hepatic artery, superior mesenteric artery and/or celiac trunk, or unreconstructible involvement of the porto-mesenteric vein. Prior to any treatment, the diagnosis of LAPC should be confirmed using histopathological examination. This can be obtained using endoscopic ultrasound (EUS) or percutaneous using CT guidance [16,17].

For pancreatic cancer, carbohydrate antigen 19-9 (CA 19-9) is currently the only biomarker which is approved by the US Food and Drug Administration. Although not specific for pancreatic cancer, it is currently considered the most favorable biomarker for pancreatic cancer management and treatment evaluation [18]. CA 19-9 should therefore be assessed at baseline, prior (i.e., after adequate biliary drainage) and after induction chemotherapy, as previous studies have suggested that a decrease in CA 19-9 following (systemic) treatment is a useful marker for treatment success [19]. It is important to assess CA 19-9 prior to induction chemotherapy, after successful biliary drainage, as the level may be influenced by obstructive jaundice [20]. Of note, approximately 5–10% of patients with pancreatic cancer do not produce CA 19-9 due to a genetic mutation in the gene for the Lewis antigen [20]. In addition to CA 19-9, other promising biomarkers such as micro-RNAs and circulating tumor DNA are being studied to more accurately predict treatment response in (locally advanced) pancreatic cancer [21,22].

### 3.2. Diagnostic Laparoscopy

The timing of laparoscopy is topic to some debate. Some centers prefer to perform a diagnostic laparoscopy prior to start of chemotherapy to optimally stage and inform patients. This procedure can be combined with the placement of a mediport, to facilitate intravenous chemotherapy administration. A diagnostic laparoscopy can also be performed prior to surgical exploration or prior to local ablative therapy. The disadvantage of the late laparoscopy is that small liver or other lesions, which reacted well to chemotherapy could not be detected anymore, which leads to inadequate staging.

A diagnostic laparoscopy is useful since 20–30% of patients with LAPC will have occult peritoneal metastases [23,24]. In these patients, local ablative therapy (aiming for local control) is less likely to be beneficial, because most patients will die from peritoneal or liver disease. During laparoscopy, the peritoneum, the liver, and lesser omentum are inspected [25]. Suspect lesions should be biopsied and pathologically assessed for tumor metastasis.

In patients where a resection is considered after induction therapy, most surgeons perform also a diagnostic laparoscopy directly prior to resection, however no data are available about the yield of this procedure.

### 3.3. Biliary Drainage

FOLFIRINOX chemotherapy requires a serum bilirubin level below 1.5 times the upper limit of normal. Therefore, patients should have adequate biliary drainage, preferably via a self-expanding metal stent. It has been demonstrated that patients with metal stents are better palliated and have fewer complications such as stent occlusion or migration when compared with plastic stents [26]. These stents can easily be replaced for plastic stents if required for ablation or removed during surgery. Percutaneous biliary drainage (PBD) can be performed as an alternative to stenting, but should only be considered if endoscopic stenting is deemed not feasible [27]. In case endoscopic drainage and PBD fail, a surgical biliary bypass (i.e., hepato-jejunostomy) can be considered. However, in the current era of metal stents, a surgical hepato-jejunostomy is required only in a minority of patients.

### 3.4. Induction Chemotherapy

FOLFIRINOX therapy is reserved for patients with good performance status (World Health Organization performance score 0–1) and adequate biliary drainage. According to the NCCN criteria [15], induction chemotherapy is advised for 8–12 cycles (4–6 months), which consists of a 2 weekly schedule of 2 h intravenous infusion of oxaliplatin 85 mg/m^2^, followed by a 2 h intravenous infusion of folinic acid 400 mg/m^2^ concomitantly with 90 min of intravenous infusion of irinotecan 180 mg/m^2^, subsequently followed by 5-FU 400 mg/m^2^ as a bolus and 2400 mg/m^2^ as a 46 h continuous intravenous infusion, with ondansetron and dexamethasone with each cycle as routinely emesis prophylaxis [28]. Consensus on the optimal period of induction chemotherapy is lacking. Most centers would advise a period of 4–6 months [6]. During FOLFIRINOX treatment, serum platelets, neutrophil count, liver and renal function should regularly be monitored. Dose reductions are allowed and very common in case of toxicity or at the patient’s request, since previous studies have suggested that the effects of FOLFIRINOX seem maintained even after nearly routine dose reductions [5,29]. Preferably, the doses of irinotecan or oxaliplatin are reduced with 25–50% of the original doses, or the 5-FU bolus is omitted. Granulocyte colony-stimulating factor (G-CSF) can be administered to prevent neutropenia [28].

In patients who are expected not to tolerate FOLFIRINOX or who progress under this treatment, gemcitabine-nab-paclitaxel (gem-nab) is generally advised [30]. However, some centers prefer gem-nab as first-line treatment over FOLFIRINOX, as this regimen is generally better tolerated [31]. Although randomized trials comparing FOLFIRINOX with gem-nab are lacking, a comparative study comprising 193 patients suggested that survival after FOLFIRINOX is superior to after gem-nab [32]. The multicenter, randomized NEOLAP trial will aim to validate these findings (Table 1). The regimen of nab-paclitaxel consists of a 30 to 40 min intravenous infusion of nab-paclitaxel at a dose of 125 mg per square meter, followed by an infusion of gemcitabine at a dose of 1000 mg per square meter, on days 1, 8, and 15, every 4 weeks.

### 3.5. Restaging after Chemotherapy

After 4–6 months of FOLFIRINOX chemotherapy, restaging is performed according to the RECIST 1.1 criteria upon contrast-enhanced CT imaging (ceCT) [11]. According to the RECIST definitions; RECIST progression requires at least a 20% increase in the sum of the tumor diameters in three directions (and an absolute increase >5 mm) or the occurrence of new lesions. RECIST partial response (i.e., regression) requires tumor shrinkage of at least 30%. RECIST stable disease is defined by the absence of progression and regression, respectively. After induction treatment with FOLFIRINOX, RECIST stable disease is mostly observed (60%), followed by RECIST progression in 20–30% and RECIST partial response in 10–20% [40]. Also, CA 19-9 should be measured at restaging. Several studies have confirmed the importance of serum CA 19-9 assessment before and after chemotherapy [41,42]. One prospective study found a 90% sensitivity for resection when there was a 30% decrease of CA 19-9 following induction chemotherapy [42]. An increase in CA 19-9 is a poor prognostic factor. It should probably prohibit surgery and lead to a switch in chemotherapy [20,42]. Per two months chemotherapy, a high-quality 2-phase (i.e., portal-venous and late arterial) CT chest–abdomen and serum CA 19-9 assessment is advised [15].

### 3.6. Selection for Surgery

In high-volume centers for pancreatic surgery, explorative laparotomy could be performed in patients with RECIST nonprogressive (stable or regression) disease after 2–4 months chemotherapy to assess the feasibility of a resection. CT imaging may underestimate the response of FOLFIRINOX or gem-nab, because discrimination between fibrosis and viable tumor is impossible [10]. As a result, a substantial proportion of patients with borderline resectable/LAPC can undergo a radical resection despite persistent apparent vascular tumor abutment or encasement on CT imaging [43]. However, criteria for resectability of LAPC are not standardized. With the use of more effective neo-adjuvant treatments, more patients with LAPC undergo a radical resection, often with vascular resections [44,45,46,47,48].

Some centers, such as the Johns Hopkins Hospital routinely perform SBRT in LAPC patients prior to surgical exploration. This was based on the series of Gemenetzis et al., suggesting that SBRT improves the probability of a radical resection in patients with LAPC and borderline resectable pancreatic cancer after induction FOLFIRINOX chemotherapy [49]. In addition, this series reported an impressive 10% complete pathological response after eight cycles of FOLFIRINOX and radiotherapy, which was associated with a median overall survival over 60 months [49].

### 3.7. Local Ablative Therapy

Patients with persistent locally advanced disease, who are in good clinical condition (WHO PS 0–1) and RECIST stable disease after 2–4 months chemotherapy can be considered for local ablative treatment if certain criteria are met. Eligibility criteria for the varying modalities are mainly dependent on the tumor orientation in relation to the surrounding structures. The most investigated techniques are SBRT, IRE, and RFA. Although randomized, controlled trials confirming the added value of local ablative modalities are currently lacking, previous cohort series report on a possible survival benefit of local ablation in combination with systemic treatment. Until randomized trials confirm the benefit of these techniques in LAPC, it is strongly advised to perform these treatments only within the setting of clinical trials. In addition to a local effect, there is increasing evidence that local ablative therapies are also capable of inducing a systemic anti-tumor response (i.e., abscopal effect) [50,51,52]. This has prompted the interest in combing local ablative modalities with immunotherapy to enhance the immunomodulatory effects of these techniques. Although clinical data are lacking, some translational studies suggest an increased efficacy when combining local ablation with immunotherapy [52,53]. High-intensity focused ultrasound (HIFU) is not considered in the current review given the limited available data on oncological outcomes, but may be a promising technique with regards to pain relief [54].

#### 3.7.1. Irreversible Electroporation (IRE)

The concept of IRE is that tumor cells are destructed by permeabilization of cell membranes through high-voltage electrical pulses [55,56,57,58,59,60]. This technique is supposedly nonthermal, and therefore causes apoptosis rather than necrosis [61,62]. IRE can be performed during open surgical exploration or percutaneous as standalone procedure with CT guidance [63,64]. For IRE, 2–6 electrodes are typically placed around the tumor, with a maximum spacing of 2.0–2.5 cm, using image guidance. Electric pulses are delivered between the electrode pairs, which disrupt the tumor cells’ membranes and hence cause cell death [63]. Patients with persistent locally advanced disease after chemotherapy who are not candidates for surgical exploration (i.e., complete 360 degrees arterial encasement or extensive involvement of multiple arterial structures) could especially benefit from a less invasive, percutaneous approach [64,65]. One may consider to perform a staging laparoscopy prior to percutaneous IRE in order to rule out occult metastatic disease, which is present in 15–25% of patients with LAPC after induction systemic treatment [24]. However, it currently is unknown whether these patients do not benefit from local ablative treatment. There is growing evidence that local ablative modalities such as IRE may induce a systemic anti-tumor response and hence, contribute also to distant disease control [52].

Based on case reports, it is advised to exclude patients with a combined severe stenosis of the common hepatic artery and main branch of the portal vein from IRE treatment, given the 10% risk of an acute portal vein occlusion, potentially resulting in liver failure and death [40]. This also accounts for patients with irreversible bleeding disorders, epilepsy, or any other unstable condition that provokes the inability to undergo general anesthesia. Patients with a past medical history of cardiac disease (i.e., cardiac arrhythmia, implantable cardioverter defibrillator, pacemaker) are preferably not treated with IRE, given the risk of inducing cardiac arrhythmias when applying the electrical pulses.

For IRE, as compared with RFA, the involvement of vital structures is less of an issue [66], but the treatment efficacy is hampered by increasing tumor size, and the procedure becomes more challenging with increasing electrode numbers. It is therefore advised to limit IRE to a maximum tumor diameter of 5.0 cm [67]. Moreover, an experimental study demonstrated that the presence of metal stents does not lead to an increase in thermal damage or the inability to produce a stable current when performing IRE. However, redirection of the electric field can cause insufficient ablations, resulting in a rim of vital tissue surrounding the metal stents [68]. These conclusions are, however, based on a very limited number of experiments, and therefore as a precaution, removal of the metal stent prior to IRE is preferred, and the same holds for RFA. Previous cohort studies report heterogeneous outcomes, with median overall survival rates varying between 15–32 months when combining IRE with systemic treatment. The current largest available series, comprising 200 patients with LAPC treated with open IRE after induction chemotherapy reports a 24.9 months median overall survival, with an 18.5% complication rate and 2% mortality [63]. In general, major complications (e.g., portal vein thrombosis, bleeding, duodenal perforation) are reported in 0–30% of patients, with mortality rates ranging between 0–11% [69,70].

#### 3.7.2. Radiofrequency Ablation (RFA)

RFA relies primarily on thermal damage to cause coagulative necrosis of tissue. RFA is mostly performed during laparotomy, using duodenal cooling and minimizing the risk of thermal damage to vital structures, but can also be performed percutaneously or using endoscopic ultrasound [71]. Additionally, small liver or peritoneal metastases can be excluded using laparoscopy immediately prior to laparotomy. Typically, RFA is used for tumor debulking (i.e., cytoreductive treatment) rather than complete ablation, because it requires a safety margin of at least 5 mm from the ablation zone to avoid thermal damage to vital structures [72]. This technique may therefore be contra-indicated in relatively small tumors with a perivascular growth pattern. According to current literature, RFA is reported as a relatively safe technique, with morbidity varying between 0–28% across studies and an RFA-related 30 day mortality rate of 0–3% [70]. Current available cohort studies report on a median overall survival ranging between 19.0 and 25.6 months when combining RFA with chemotherapy [70]. The results of the ongoing multicenter, randomized controlled PELICAN trial in the Netherlands (NTR5517), which compares chemotherapy and RFA with chemotherapy alone are expected in 2022 [73]. The PELICAN trial aims to determine whether there is a survival benefit of RFA in patients with nonprogressive LAPC after two months of induction chemotherapy (Table 1).

#### 3.7.3. Stereotactic Body Radiation Therapy (SBRT)

SBRT is a noninvasive ablation modality, which uses a hypofractionated, high dose of radiation to achieve cell death [74]. For SBRT, tumor size does not necessarily hamper treatment efficacy, but careful treatment planning is warranted in the case of small bowel, duodenal, or gastric tumor involvement due to the risk of gastro-intestinal toxicity. Previous studies on the safety and efficacy of SBRT in LAPC report on a 0–28.4% risk of acute complications (i.e., within 3 months after SBRT) and 0–13% late complications (i.e., >3 months after SBRT), with median overall survival ranging between 10–20 months [70]. For patients at risk of toxicity, fractionated delivery regimens and prophylactic use of proton-pump inhibitors are used to minimize morbidity [75]. The currently ongoing randomized CROSSFIRE trial in the Netherlands (NCT02791503) compares overall survival between percutaneous IRE and stereotactic radiation therapy in patients with LAPC (Table 1).

When following the above-mentioned criteria, some tumors may particularly be candidates for IRE, SBRT, or RFA, but in some cases, multiple modalities may be feasible. Since there are currently no published phase III randomized controlled trials with IRE, SBRT, or RFA in LAPC (Table 1), the choice of treatment if a patient meets the selection criteria for more than one modality will primarily depend on the experience/preference of the treating physician. It should be noted that no benefit in overall survival has been demonstrated in an RCT for any of the ablative treatments. Therefore, we recommend to perform ablative techniques only in clinical trials.

### 3.8. Surgical Procedure

In clinical practice, a staging laparoscopy is frequently performed prior to surgical exploration. In case no metastases are found, a laparotomy is performed whereafter the peritoneum, liver, and abdominal cavity are (once more) assessed for the presence of metastases.

When no metastases are found, a high-quality intra-operative ultrasound (IOUS) may demonstrate more often (borderline) resectable disease compared with ceCT [76]. IOUS is performed in many centers worldwide before dissection, either by an experienced (interventional) radiologist or by the surgical team. This is commonly an interventional radiologist. For IOUS, state-of-the-art ultrasound hard- and software are necessary. This includes a high-frequency linear probe which is placed directly on the pancreas or on the surface of the stomach for a transgastric approach. Using a structured assessment with ultrasound, the largest tumor diameters in three directions and extent of tumor involvement with the porto-mesenteric vein, celiac trunk, hepatic, and superior mesenteric artery (SMA) are documented.

When IOUS reveals potentially resectable disease in the head of the pancreas, an artery-first approach is usually advised, although numerous approaches are available and may be used as the local situation dictates [77]. Treitz ligament is mobilized, and the left side of the SMA sheath is exposed. Next, the omental bursa is opened, and a wide Kocher maneuver is performed, and Catell–Braasch maneuver when an SMV/PV reconstruction is required [78]. The SMA base and right side of the SMA sheath are exposed. From here, an uncinate-first approach [79] or other “artery-first approach” to resection can be used. The uncincate-first approach includes a retrograde resection of the pancreatic head, starting with the division of the proximal jejunum, lateralization of the uncinate process to the right side, followed by dissection of the pancreas from the retroperitoneum and the mesenteric root [79]. For an artery-first approach, the left renal vein can be identified after mobilization of the right colonic flexure and used as a guide to identify the origin of the SMA coming from the aorta [80]. Frozen sections are taken in case of presumed arterial abutment (e.g., tumor tissue left of the SMA). Suspect aorto-caval lymph nodes are also sent for frozen section to rule out extraregional nodal metastases. In case of disseminated disease, a resection or local ablation is considered not to be in the patient’s best interest [81]. For pancreatic head tumors, as mentioned previously, a palliative gastro-enterostomy may be considered in locally advanced patients with (potential) gastric outlet obstruction.

**Figure 1:**
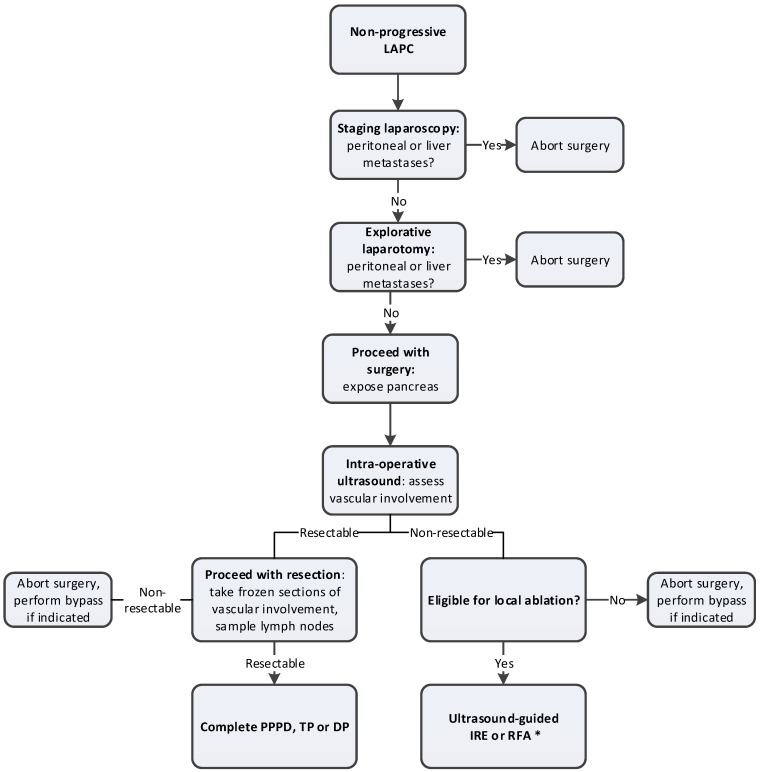
Overview of surgical procedure of LAPC after induction chemotherapy. * Since randomized studies confirming the efficacy of ablation in LAPC are lacking, patients should preferably be treated within the context of clinical trials; LAPC: locally advanced pancreatic cancer; PPPD: pylorus-preserved pancreatoduodenectomy; TP: total pancreatectomy; DP: distal pancreatectomy; IRE: irreversible electroporation; RFA: radiofrequency ablation.

If arterial involvement is <180°, a resection can often be performed [63]. In case of pancreatic body cancer with involvement of the celiac trunk, a distal pancreatectomy with celiac artery resection (DP-CAR or Appleby procedure) may be performed. We previously described the details of this procedure elsewhere [82]. DP-CAR combined with (neo)adjuvant therapy is associated with an 18–19 months overall survival, according to a recent systematic review and retrospective international series [83,84]. Overall, 10–15% of allcomers may become eligible for a curative-intent resection after induction chemotherapy [40]. For patients who are fit to undergo FOLFIRINOX chemotherapy, the cumulative proportion of patients undergoing resection after 2–4 months chemotherapy is 25–30% [6]. The surgical procedure for LAPC after induction chemotherapy is summarized in Figure 1.

### 3.9. Postoperative Care

Postoperative care of patients following resection or ablation in LAPC does not differ much from the standard of care following pancreatic surgery. Postoperative imaging may be indicated if patients present with a postoperative fever, severe pain, or increasing inflammatory markers to detect possible complications such as pancreatic fistulae, fluid collections, and bleedings. Standard follow-up consists of three-monthly CT imaging assessment, according to RECIST, and adjuvant chemotherapy, similar to the induction scheme. In this aspect, monitoring of CA 19-9 response may detect early tumor recurrence or disease progression [20]. Plastic stents are preferably replaced by metal wall stents, if still present. Stent revisions may be needed postoperatively if the patient presents with clinical signs of cholangitis (i.e., obstructive jaundice, fever).

Data on the efficacy of adjuvant chemotherapy after resection or ablation in LAPC are scarce. Most centers complete treatment up to a total of 8–12 cycles of FOLFIRINOX (including preoperative cycles, but strong evidence is lacking.

**Figure 2:**
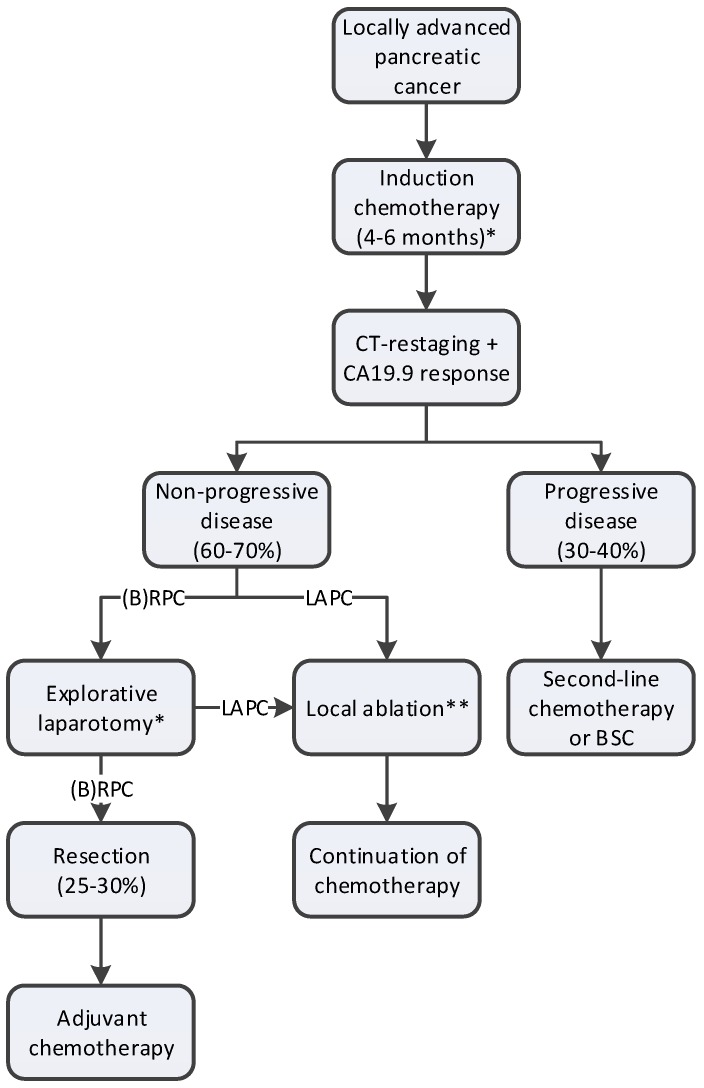
Proposed work-up and treatment plan of LAPC. * Diagnostic laparoscopy can be performed either prior to the start of induction chemotherapy or prior to explorative laparotomy; ** Since randomized studies confirming the efficacy of ablation in LAPC are lacking, patients should only be treated within the context of clinical trials; BSC: best-supportive care; LAPC: locally advanced pancreatic cancer; (B)RPC: borderline resectable pancreatic cancer.

A proposed work-up and treatment plan of LAPC is depicted in Figure 2.

## 4. Future Directions on the Management of Locally Advanced Pancreatic Cancer

A summary of the available evidence and targets for future research regarding the induction treatment, response evaluation and local ablative strategies for LAPC is summarized in Table 2.

## 5. Conclusions

The treatment of LAPC has greatly evolved in recent years. Systemic treatment with FOLFIRINOX and gemcitabine-nab-paclitaxel alone leads to a 14–16 months survival, whereas in patients with stable disease after 2–4 months systemic treatment, based on imaging and CA 19-9, resection is feasible in 25–30% (10% of all-comers), with a median 30 months overall survival. The criteria for surgical exploration and ablation of LAPC are not yet standardized. Future studies will have to identify the clinical relevance of biomarker (e.g., circulating tumor DNA, inflammatory parameter risk scores) stratified treatment of patients with LAPC. Due to the improvement of systemic therapy, outcomes of (surgical) treatment of LAPC have much improved. If resection is not feasible, IRE, RFA and SBRT could be used, but only in the setting of clinical trials. Interpretation of the impact of FOLFIRINOX and/or gem-nab is difficult and requires expertise and patient participation in prospective cohorts, and preferably randomized controlled trials. International collaboration is required to further improve the management, survival, and quality of life of patients with LAPC.

## Figures and Tables

**Table 1:** Registered randomized phase II/III trials in locally advanced pancreatic cancer comprising >100 patients.

Registry Number	Acronym	Phase	Control	Interventions	Primary Outcome	Sample-Size	Centers	Country
NCT01827553 [33]	CONKO-007	III	Chemotherapy alone	Chemoradiation + chemotherapy	OS	830	Multicenter	Germany
NCT02125136 [34]	NEOLAP	II	Gemcitabine-nab-paclitaxel	FOLFIRINOX	Resectability	168	Multicenter	Germany
NCT03377491 [35]	PANOVA-3	III	Gemcitabine–nab-paclitaxel	Gemcitabine–nab-paclitaxel + NovoTTF-100L(P)	OS	556	Multicenter	Austria, Canada, France, Italy, Spain, Switzerland, USA
NCT02806687 [36]	THERGAP-02	II	Gemcitabine	Gemcitabine + CYL-02 injection	PFS	100	Multicenter	France
NCT02791503 [37]	CROSSFIRE	III	Chemotherapy + SABR	Chemotherapy + IRE	OS	138	Multicenter	The Netherlands
NCT01926197 [38]	PANC0015	III	mFOLFIRINOX	mFOLFIRINOX + SBRT	PFS	172	Multicenter	Canada, USA
NTR5517 [39]	PELICAN	III	Chemotherapy only	Chemotherapy + RFA	OS	228	Multicenter	The Netherlands

Registered active, randomized, phase II/III interventional studies in LAPC (source: clinicaltrials.gov. and Netherlands Trial Registry, access date 07/01/2019). mFOLFIRINOX: modified FOLFIRINOX; IRE: irreversible electroporation; RFA: radiofrequency ablation; SBRT: stereotactic body radiation therapy; SABR: stereotactic ablative radiotherapy; OS: overall survival; PFS: progression-free survival; LAPC: locally advanced pancreatic cancer.

**Table 2:** Future directions on the management of locally advanced pancreatic cancer.

Subject	Current Evidence	Future Directions
Chemotherapy	Promising results are reported for the use of FOLFIRINOX and gemcitabine-nab-paclitaxel as induction treatment of LAPC. It currently remains unknown which of the two regimen is superior as first-line treatment of LAPC.	The results of the ongoing NEOLAP study will assess the superiority of either FOLFIRINOX or gemcitabine-nab-paclitaxel as induction treatment for LAPC.
	There currently is no consensus on the optimal duration of induction treatment of LAPC. Some centers advocate two months treatment duration, whereas other centers routinely treat patients for four months. This also accounts for the use of adjuvant chemotherapy after initial induction treatment	Future studies should also determine the optimal duration of induction chemotherapy and added value of adjuvant chemotherapy for LAPC after resection.
Response evaluation	Current imaging modalities often underestimate the response of LAPC to induction chemotherapy, as they cannot distinguish fibrosis from vital tumor tissue. Biomarkers may aid in selecting patients with good overall response to chemotherapy but lack specificity.	New response evaluation criteria and/or imaging modalities are required to more accurately determine resectability after induction chemotherapy.
		The added value of biomarkers to predict response to induction chemotherapy should be established in future studies.
Surgery	Current evidence on resection of LAPC after induction chemotherapy is promising, but randomized trials confirming the additional value of surgery after chemotherapy are lacking.	Future randomized trials should establish the added value of surgery compared with that of chemotherapy alone in LAPC.
	Because of the lack of accuracy of current imaging modalities to predict resectability of LAPC, several centers advocate routine surgical exploration in patients with at least stable disease after induction chemotherapy.	The optimal selection criteria for surgical exploration of LAPC after induction chemotherapy should be established.
Neoadjuvant radiotherapy	Some centers perform routine SBRT of LAPC prior to surgical exploration after induction chemotherapy to improve the chance of a radical resection, decrease local recurrence, and improve OS. The added value of this approach has not yet been determined.	The added value of SBRT prior to surgical exploration should be compared with that of chemotherapy alone in patients with LAPC undergoing surgical exploration after induction chemotherapy.
Ablative therapies	Local ablative therapies are considered in some centers in patients with persistent LAPC after induction chemotherapy. Randomized trials to determine the added value of ablative therapies to chemotherapy-alone are lacking.	The results of the currently ongoing PELICAN trial will assess the added value of RFA to chemotherapy-alone.
	Currently there are no completed trials comparing multiple ablative modalities. Therefore, the superiority of either technique (IRE, RFA, SBRT) remains unknown.	The ongoing CROSSFIRE trial will determine the superiority of either IRE or SABR in patients with LAPC after induction chemotherapy. Future comparative studies are needed to determine the most effective local ablative treatment in LAPC.
	There is increasing evidence that local ablative therapies can induce a systemic anti-tumor response (i.e., abscopal effect). It is suggested to combine local treatment with immunotherapy to both increase local-and distant disease control	Future studies should focus on combining local ablative therapy with (systemic) immunotherapy.

LAPC: locally advanced pancreatic cancer; IRE: irreversible electroporation; RFA: radiofrequency ablation; SBRT: stereotactic body radiation therapy; SABR: stereotactic ablative radiotherapy.

## Appendix A. Search Strategy: PubMed

((locally advanced pancreatic cancer OR LAPC OR locally advanced PDAC OR non-resectable pancreatic cancer OR unresectable pancreatic cancer [tiab]) AND (induction OR neoadjuvant OR pre-operative OR preoperative [tiab]) AND (chemotherapy OR chemo OR FOLFIRINOX OR gemcitabine [tiab])).
